# Save Your Children

**Published:** 1873-12

**Authors:** 


### SAVE YOUR CHILDREN.

A YOUNG man of twenty-one fell down dead the other day—an only son of one of the wealthiest and most aristocratic of the New York Knickerbockers; he died of *delirium tremens*, a besotted drunkard! His accomplished father gave him an annual "allowance," and permitted him to grow up without any profession or calling. Supply an idle young man with money and his ruin is a certainty. Our inebriate asylums are tenanted by the sons of men of wealth, position and cultivation. The first step towards making a man of your son is to train him to earn what he spends. The next best step is to teach children to save money, beginning at the age of three years. Where there is a family of children it might be well to offer a premium to the one who saved the most during the year. Show them the advantage of having money at interest, and of having money in bank; that every dollar at interest is seven cents more at the end of the year, and in about ten years it grows to two dollars. Explain to them how, by having money in a savings bank, bearing interest, that it not only increases, but that by having it where it can be obtained any day, opportunities are constantly occurring where things can be purchased at a very low rate for cash in hand, and that always purchases can be made to greater advantage by paying for them at the time in money.

Children are naturally imitative and ambitious to excel—to get beyond others—the efforts, for example, which boys and girls have made within a few years to obtain the greatest variety of postage stamps for their albums. Once get a child to save money for good purposes and he is safe for all time; for while he is practising accumulation he is at the same time learning useful lessons of economy and self-denial, to say nothing of using time in such a way as to gain something by it.

Any reflecting person must know that the institution of savings banks has had a wonderful effect, all over the country, in proportion as persons are intelligent and industrious, to stimulate an ambition to increase bank balances, they being looked upon with the greatest respect and deference who have the largest sums laid by.

Teach the young, by showing it to them in various ways, that persons who have money on hand command the consideration of their neighbors, and have a feeling of self-respect, elevation and independence to which those who have no money on hand are perfect strangers.

It is to the habit of taking care of their money, and the corresponding virtue of habitual economies and self-denial on the part of Germans who come to this country, that they are everywhere thrifty, and are rapidly becoming the owners of corner lots, of the best places of business, whether in Wall street or Broadway. The proportion of German importing houses and of German bankers is constantly increasing, and so are their shipping lines, and wholesale establishments in every variety of business. Let American parents ponder this.

In the City of Ghent, Belgium, without any law, merely through the instrumentality of school teachers, who have had a wise and conscientious interest in the welfare of those committed to their care, five sevenths of the children have become depositors in the savings banks, bringing their cents and half cents at a time. Of 7,983 boys and girls in the primary schools, 7,583 have "bank accounts," amounting to $54,920. Of the three thousand and thirty-nine little ones at the infant schools nineteen hundred and twenty are depositors, with thirteen thousand two hundred dollars to their credit; these are among the poorer classes, who do not pay for instruction. Of the thousand and seventy-nine scholars who pay for their schooling, six hundred and forty had deposited $4.537. The curious fact is here seen, that while a little more than half the scholars who were better off saved their money, nine tenths of the poorer classes became depositors, showing that the richer children were less inclined to save than the poorer.

A lady came to me the other day and said that all her husband had to eat that morning for breakfast was a penny roll of bread. A few years ago they drove a carriage, owned a house in Washington square, and had a country seat on the Hudson. Mother, the next time you meet your children at the dining table, look at your bright eyed daughter and think of her begging a slice of bread after you have been laid in your grave; and that merry looking son, with his clear, smooth face and high forehead, and countenance all beaming with the confidence and hope of youth; and what if he should come to want, and be glad to get a night's lodging on a bench at the police station? To prevent it give each one of them a "job" on the instant, by which they can fairly earn a dime or two, and to-morrow go down to the "Dime Savings Bank," in Canal street or Astor place, and open an account for them.

Fairly earn was purposely written. To give a child, or any one else, more for doing their work than it is worth, lays the foundation for injustice, extravagance and beggary. Such persons soon get into the habit of asking more for their work than it is worth; employers will not engage them; they have nothing to do, and must borrow, cheat, beg or steal, or get discouraged and drink, and die forty years before their time.

About one quarter of the population of New York city is of German descent. You will see ten Americans lounging around street corners in idleness and dissipation where there is one German, because the latter will work for half price rather than get nothing—will work for his board rather than be idle and take the money he has in his pocket to pay for it, and will continue to work until he can get better wages. An American youth demands full pay or nothing, and is half his time out of employment, consequently does not get ahead—"lives from hand to mouth," and his life becomes a failure.

But, mother dear! while you are teaching your loved ones to save their money, impress upon their young minds that there are two uses for it—the first, to provide for their own wants; the second, a higher and nobler, to use a portion of what is not personally needed for helping others—for the poor, the sick, the disabled and unfortunate; and another portion to prevent poverty, and sickness, and misfortune, by promoting education, encouraging industry and economy, building churches, establishing and maintaining

Sunday schools, Bible classes, tract distribution, and whatever well devised and well working scheme of charity or benevolence may present itself; to do a little for every one, and thus get into such a habit of giving to do good that it is done without an effort—spontaneously, instinctively, with absolute pleasure, and never be bothered with the thousand and one quandaries and self-questionings which so afflict the greedy and narrow minded as to whether they can afford to give a dollar—giving one with the agony of a drawn tooth. Then clinch all four teachings with the blessed announcement, "He that giveth to the poor lendeth to the Lord."

